# Enxerto Multiarterial na Cirurgia de Revascularização Coronária. A Busca Renovada por Resultados Aprimorados

**DOI:** 10.36660/abc.20230094

**Published:** 2023-03-22

**Affiliations:** 1 Universidade Federal de São Paulo – UNIFESP Escola Paulista de Medicina e Hospital São Paulo Disciplina de Cirurgia Cardiovascular São Paulo SP Brasil Disciplina de Cirurgia Cardiovascular – Escola Paulista de Medicina e Hospital São Paulo – Universidade Federal de São Paulo – UNIFESP, São Paulo, SP – Brazil.

**Keywords:** Revascularização Miocárdica/enxerto, Revascularização Miocárdica/tendências, Artéria Torácica Interna/cirurgia, Doença da Artéria Coronariana/cirurgia, Fatores de Risco/complicações

Com uma crescente série de evidências contemporâneas e históricas fortalecendo a cirurgia de revascularização miocárdica (CRM) como o procedimento mais eficaz para o tratamento da doença arterial coronariana (DAC) aterosclerótica avançada, a busca por melhores resultados e aprimoramento da qualidade está se acelerando.^1^

A patência do enxerto a longo prazo é fundamental para os benefícios da cirurgia, evitando o desencadeamento de infarto do miocárdio (IM) espontâneo e aumentando a sobrevida a longo prazo, o que é demonstrado com o emprego de condutos da artéria torácica interna (ATI) anastomosados à artéria coronária descendente anterior esquerda.

Uma vez que os enxertos arteriais demonstraram superioridade sobre os enxertos convencionais de veia safena (EVS) em termos de perviedade a longo prazo, torna-se intuitivo empregar outros enxertos arteriais para benefício incremental.

Isso se alinha e apoia a compreensão contemporânea da fisiopatologia da DAC, onde o conceito desatualizado, mas ainda vigente, de isquemia miocárdica crônica como a principal causa de resultado adverso na DAC foi sepultado.A ruptura e a erosão da placa associada à estenose não crítica, comumente localizada na artéria coronária, longe da placa estável, são as causas da maioria dos infartos do miocárdio, com a evidência da carga aterosclerótica como o principal determinante dos resultados em DAC.^2^ A CRM anastomosa um enxerto coronário à porção distal do vaso doente, contornando suas numerosas placas ateroscleróticas espalhadas a montante que formam o substrato para ruptura da placa, trombose e infarto do miocárdio, em última análise, levando à morte e insuficiência cardíaca.

Paredes et al.,^3^ relatam os resultados precoces de pacientes submetidos à CRM com múltiplos enxertos arteriais (MEA) em comparação com um único enxerto arterial (UEA) em um estudo de coorte do REPLICCAR II (Registro Paulista de Cirurgia Cardiovascular II). De uma coorte original de 3.122 pacientes, 531 (17%) receberam múltiplos enxertos arteriais. Após pareamento pelo escore de propensão, os pacientes do grupo UEA apresentaram maior prevalência do sexo masculino e história familiar de DAC, com maior taxa de procedimentos de urgência, pneumonia recente e eventos coronarianos agudos. Em contraste, os pacientes do grupo UEA eram mais velhos e apresentavam maior taxa de diabetes mellitus, hipertensão, infarto do miocárdio prévio e tabagismo.^3^

Reiterando achados de relatórios anteriores, o uso de MEA foi associado a uma taxa mais alta de infecção profunda da ferida esternal (IPFE). Não foram observadas diferenças estatisticamente significativas com acidente vascular cerebral pós-operatório, lesão renal, tempo de intubação, mortalidade e tempo de internação > 14 dias. É digno de nota que a taxa de mortalidade hospitalar foi de 1,8% em ambos os grupos, tornando-se uma conquista notável em uma população com DAC avançada, favorável em comparação com os números de um banco de dados nacional brasileiro, o registro BYPASS.^4^

Embora seja instintivo que os enxertos de múltiplas artérias possam fornecer perviedade superior a longo prazo e resultados clínicos em comparação com o EVS, as evidências permanecem controversas e a prova de conceito está faltando até o momento. Vários estudos observacionais e meta-análises sugeriram a associação de múltiplos enxertos arteriais com benefícios superiores de sobrevida a longo prazo.^5 , 6^ No entanto, o Arterial Revascularization Trial (ART), o único estudo randomizado a comparar pacientes recebendo enxertos bilaterais versus únicos de ATI, não encontrou nenhuma diferença significativa na taxa de sobrevida de 10 anos.^7^ No entanto, uma análise post-hoc do estudo ART sugeriu um benefício do enxerto de ATI bilateral em relação ao enxerto de ATI^8 , 9^ único quando a análise de intenção de tratar foi restrita a pacientes com idades entre 50 e 70 anos, com uma incidência significativamente menor de eventos adversos maiores (mortalidade por todas as causas.

Uma preocupação de segurança dos achados do REPLICCAR II está relacionada à taxa mais alta de IPFE, que ficou em 5,6% no braço MEA e 2,26% no braço UEA. O risco de cicatrização prejudicada pode ser minimizado com a seleção cuidadosa do paciente e modificação da técnica de dissecção da ATI para esqueletizada em vez de pediculada, o que preserva a circulação colateral e o suprimento sanguíneo esternal.^10 , 11^ Evidências do estudo ART e outros demonstraram que, quando a técnica esqueletizada é usada para a coleta de ATIB, nenhuma diferença é observada uma vez em comparação com o grupo ATI único.^12^ A taxa de esqueletização ATI de 52% no braço REPLICAR II MEA fica abaixo da margem padrão para segurança, provavelmente justificando a diferença obtida.

No entanto, além do estímulo para o uso de MEA em CRM, o EVS também está fazendo progressos significativos em resultados em longo prazo. A técnica no-touch de dissecção de EVS, que envolve a remoção de um EVS pediculado com o tecido perivascular intacto sem manipulação direta ou distensão de alta pressão, preservando a integridade do endotélio e da parede do vaso, demonstrou melhora da perviedade do conduto VS a longo prazo, comparável à perviedade do enxerto de ATI.^13 , 14^

Finalmente, os autores devem ser enaltecidos pelo enorme esforço para construir o registro REPLICCAR II, fornecendo informações valiosas para os sistemas de saúde e sinalizando a prática nacional de revascularização cirúrgica coronariana.
