# Supplementary material for: Enxerto Multiarterial na Cirurgia de Revascularização Coronária. A Busca Renovada por Resultados Aprimorados
Source: Arq Bras Cardiol. 2023 Mar 22;120(3):e20230094. [Article in Portuguese] doi: 10.36660/abc.20230094 (PMC10399962; doi:10.36660/abc.20230094)
Supplement: Translation

# Translation: Multi-Arterial Grafting in Surgical Coronary Revascularization. The Renewed Quest for Enhanced Outcomes

**Affiliations:** 1 São Paulo SP Brazil Disciplina de Cirurgia Cardiovascular – Escola Paulista de Medicina e Hospital São Paulo – Universidade Federal de São Paulo – UNIFESP, São Paulo, SP – Brazil.

**Keywords:** Myocardial Revascularization/transplant, Myocardial Revascularization/trends, Mammary Arteries/surgery, Coronary Artery Disease/surgery, Risk Factors/complications

With a large body of contemporary and historic evidence strengthening coronary artery bypass surgery (CABG) as the most effective procedure for treating advanced atherosclerotic coronary artery disease (CAD), the quest for enhanced outcomes and quality improvement is steadily underway.^1^

Long-term graft patency is crucial for the benefits afforded by surgery, averting spontaneous myocardial infarction rate (MI) and increasing long-term survival, which is demonstrated with the employment of internal thoracic artery (ITA) conduits anastomosed to the left anterior descending coronary artery.

Since arterial grafts have demonstrated superiority over conventional saphenous vein grafts (SVG) in terms of long-term patency, it becomes intuitive to employ other arterial grafts for incremental benefit.

It aligns with and supports the contemporary understanding of CAD pathophysiology, where the outdated but still-running concept of chronic myocardial ischemia as the leading cause of adverse outcome in CAD have been laid to rest. Plaque rupture and erosion associated with noncritical stenosis commonly located in the coronary artery away from the stable plaque are causatives of most MI, with the evidence of the atherosclerotic burden as the primary determinant of outcomes in CAD.^2^CABG attaches a coronary graft to the distal portion of the diseased vessel bypassing its numerous atherosclerotic plaques scattered upstream that form the substrate for plaque disruption, thrombosis, and MI, ultimately leading to death and heart failure.

Paredes et al.^3^report the early outcomes of patients who underwent CABG using multiple arterial grafts (MAG) compared to a single arterial graft (SAG) in a cohort study of the REPLICCAR II (Paulista Cardiovascular Surgery Registry II). From an original cohort of 3122 patients, 531 (17%) received multiple arterial grafting. After propensity score matching, the patients in the SAG group had a higher prevalence of males and familiar history of CAD, with an increased rate of urgent procedures, recent pneumonia, and acute coronary events. In contrast, the patients in the SAG group were older and had a higher rate of diabetes mellitus, hypertension, previous myocardial infarction, and smoking.^3^

Reiterating findings of prior reports, the use of MAG was associated with a higher rate of deep sternal wound infection (DSWI). No statistically significant differences were observed with postoperative stroke, kidney injury, intubation time, mortality, and length of hospital stay > 14 days. Of note, the hospital mortality rate was 1.8% in both groups, making it an outstanding achievement in such a population with advanced CAD, favorable compared to figures of a Brazilian national database, the BYPASS registry.^4^

Although instinctive that multiple artery grafts may provide superior long-term patency and clinical outcomes compared to SVG, evidence remains controversial, and proof of concept is lacking thus far. Several observational studies and meta-analyses suggested the association of multiple arterial grafts with superior long-term survival benefits.^5 , 6^However, the Arterial Revascularization Trial (ART), the sole randomized trial ever to compare patients receiving bilateral versus single ITA grafts, found no significant difference in the 10-year survival rate.^7^However, a post-hoc analysis of the ART trial suggested a benefit of bilateral ITA over single ITA grafting when the intention-to-treat analysis was restricted to patients between ages 50 and 70 years, with a significantly lower incidence of major adverse events (all-cause mortality, myocardial infarction, or stroke) in the BITA arm.^8 , 9^The 2018 European Society of Cardiology/European Association for Cardio-Thoracic Surgery (ESC/EACTS) Guidelines on Myocardial Revascularization recommends the consideration of a second arterial graft (RITA or radial artery) as an adjunct to LITA in appropriate patients (class IIA).^9^

A safety concern from the REPLICCAR II findings is related to the higher rate of DSWI, which stood at 5.6% in the MAG arm and 2.26% in the SAG arm. The risk of impaired wound healing can be minimized with careful patient selection and modification of the ITA dissection technique to skeletonized rather than pedicled, which preserves collateral circulation and sternal blood supply.^10 , 11^Evidence from the ART trial and others demonstrated that when the skeletonized technique is used for BITA harvesting, no difference is seen once compared to the single ITA group.^12^The 52% ITA skeletonization rate in the REPLICAR II MAG arm falls below the standard margin for safety, likely accounting for the difference attained.

However, aside from the push for MAG usage in CABG, the SVG is also making significant strides in performance. The no-touch SVG harvesting technique, which involves removing a pedicled SVG with the intact perivascular tissue without direct manipulation or high-pressure distension, preserving the endothelium and vessel wall integrity, has demonstrated improved long-term SV conduit patency, comparable to ITA graft patency.^13 , 14^

Finally, the authors should be commended for the tremendous effort to build up the REPLICCAR II registry, providing invaluable information for health systems and signaling the national practice on surgical coronary revascularization.
